# Electrical Impedance Tomography Use in Neonatal and Pediatrics Patients: A Narrative Review

**DOI:** 10.1002/hsr2.71458

**Published:** 2025-11-24

**Authors:** Xuelian Yang, Gelan Miao, Chaobing Yang, Li Liu, Xianying Lei

**Affiliations:** ^1^ Department of Intensive Care Unite the Affiliated Hospital of Southwest Medical University Luzhou China; ^2^ Department of Anesthesiology the Affiliated Hospital of Southwest Medical University Luzhou China

**Keywords:** Children, Electrical impedance tomography, Pulmonary imaging, Review, Ventilation distribution

## Abstract

**Background and Aims:**

Electrical impedance tomography(EIT) is a Noninvasive, non‐radiative, bedside imaging and monitoring tool, which has been developed for more than 40 years. With EIT used in monitoring regional lung ventilation and perfusion for many years, it is not only a new research tool different from X‐ray, CT, or ultrasound, but it also more easily provides instructions in all aspects of treatment for clinicians. Nowadays, the EIT has been used in thousands of studies in adults. EIT offers more potential significance and value in pediatric patients. As a result, we provide an overview of the data that support the clinical use of EIT in pediatric and neonatal patients (as of 2024) in this review, putting a focus on appropriate application and outlining potential future paths.

**Methods:**

As of June 2024, we identified using the following MeSH terms: EIT, infants, neonates, pediatrics, and children. The database search encompassed PubMed, Embase, and Web of Science, and we took into account only English‐language papers.

**Results:**

EIT provides valuable insights into lung function and ventilation/perfusion distribution, particularly for detecting pneumothorax. This technology allows clinicians to identify regional ventilation disparities and dynamically optimize ventilator settings, thereby enhancing mechanical ventilation strategies.

**Conclusion:**

EIT has the potential to become a ubiquitous imaging modality, however, large‐scale, multicenter investigations are essential to evaluate its clinical efficacy in critically ill pediatric patients.

## 1 Introduction

Electrical impedance tomography(EIT) is an imaging technology that applies safe current to the human body and extracts human biomedical information by using different tissues with different resistivity in different physiological and pathological states. As a Noninvasive, non‐radiative, bedside imaging and monitoring tool, EIT provides dynamic tidal images of gas distribution. Obviously, EIT is no longer a new technology, but its clinical use at the bedside is still in its primary stage in pediatric patients. For its convenient features, we conclude it can fulfill the criteria of an ideal pediatric lung monitor for the modern.

Children are not small adults, and this is particularly true when it comes to the respiratory system. In fact, children's lungs will slowly develop. The differences between a child's and an adult's airways become less obvious as they get older, and by the time a child is 6 or 8 years old, their respiratory systems are comparable [1]. Pediatric patients have lower relaxation volumes and smaller elastic retraction forces than adults, which makes them more vulnerable to airway collapse [2]. This vulnerability highlights the need for advanced visual tools to accurately assess lung conditions in children. Such tools can enable clinicians to better understand the physiological and anatomical characteristics of pediatric lung diseases and, most importantly, improve treatment outcomes. To date, the literature on EIT is expanding rapidly and new commercial devices are becoming available. A large observational study also showed that continuous monitoring of the EIT for up to 72 h is feasible and safe [3]. Therefore, an increasing use in the clinical setting can be forecasted.

Tidal volume(TV) distribution, regional compliance, and end‐expiratory lung volume(EELV) in mechanical ventilation under various circumstances were the primary evaluation indicators in earlier EIT applications for pediatric patients [4, 5, 6, 7]. A 2002 study used electrical impedance tomography to assess lung tissue resistivity as a function of frequency in 155 normal children (ages 0–3 years) and 25 preterm infants, aiding in the analysis and characterization of lung tissue maturation and growth [8]. The purpose of this study is to outline the features and clinical uses of EIT in pediatric patients, which may facilitate improved lung function monitoring during clinical practice and research planning. Table 1 provides a concise introduction to EIT for a better understanding of its principles and utility [9, 10].

**Table 1:** Brief introduction.

WHAT	Electrical impedance tomography (EIT) is a beside medical technique, it is possible to reconstruct cross‐sectional images of an object's local electrical impedance from sets of surface data.
HOW	EIT uses injection of high frequency and low amplitude electrical currents, typically through 16 or 32 electrodes around the thorax, to obtain images of a cross section of the lungs.
WHERE	16 or 32 electrodes are placed above on the fourth to the sixth ribs of the thorax 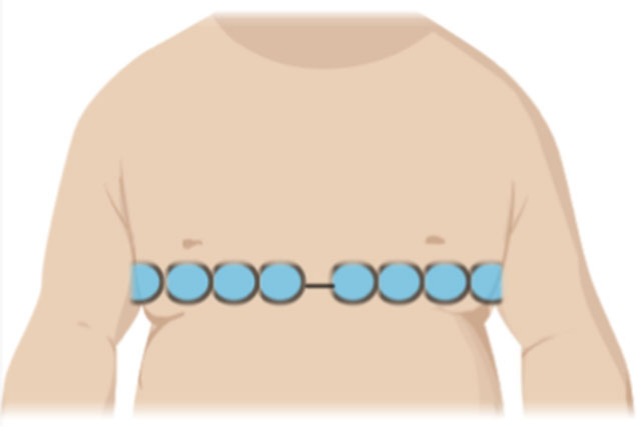
Result	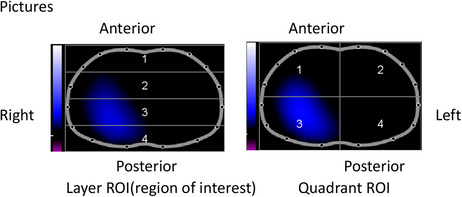
	EIT parameter
	global inhomogeneity (GI)
	end‐expiratory lung impedance (EELZ)
	Variation of impedance (∆*Z*)
	center of ventilation (CoV)
	regional ventilation delay (RVD)
	Overdistension and collapse (OD/CL)

## 2 Methods

This narrative review presents a comprehensive framework to explore the relationship between EIT and various diseases, providing insights into clinical application and future research directions. The inclusion criteria consisted of full‐text articles published in English up to June 2024. This time frame was selected to incorporate the latest developments in the field. As of June 2024, we conducted a literature search using the MeSH terms: EIT, infants, neonates, pediatrics, and children. The search encompassed PubMed, Embase, and Web of Science. Additionally, we manually reviewed the references of the retrieved articles. Exclusion criteria included nonclinical studies, articles not written in English, studies that did not report outcomes, conference papers, posters, and unpublished or non‐peer‐reviewed studies.

## 3 EIT With Lung Ventilation Without Intervention and Therapy

EIT holds significant potential as a therapeutic tool for monitoring physiological states and guiding interventions in pediatric and neonatal populations. In small observational trials, EIT has been widely utilized to characterize the clinical status of the lung in children. As early as 2009, a notable study reported both regional and global ventilation inhomogeneities in term and preterm newborns [11]. There is one study showing that the ventilation distribution in spontaneously breathing infants and children differs from previously recorded patterns. Body position‐related changes in ventilation distribution were not statistically significant in reversing the adult trend [12]. It is well understood that, after birth, a newborn transitions from receiving oxygen through maternal cord blood to obtaining oxygen through lung respiration. EIT allows for real‐time monitoring of this respiratory transition in newborns. Tingay's study further illustrates the mechanics of newborn breathing immediately following birth [13]. When children have a pulmonary infection, the ability of spontaneous cough and sputum cough is relatively weak, and chest physiotherapy can improve the ability of auxiliary sputum discharge to a certain extent. It facilitates the removal of tracheobronchial secretions, which lessens airway resistance, enhances gas exchange, and facilitates breathing [14]. To clarify the pulmonary ventilation during chest physiotherapy, monitoring of EIT may help to understand the recovery of EELV the regional resolution of EIT could be used to visualize regional airway obstruction during mechanical ventilation or spontaneous breathing [15].

## 4 EIT With Some Ventilation Mode

### 4.1 Noninvasive Respiratory Support

Over the last decades, noninvasive ventilation has become an established treatment modality in children. Most severely preterm newborns need to be supported by positive pressure ventilation either during the transitional period or at birth. In the neonatal intensive care unit, the majority of these babies still need substantial respiratory assistance since they come with respiratory distress. Additionally, it aids in the successful treatment of childhood obstructive sleep apnea syndrome. EIT has been utilized in several studies on noninvasive respiratory support (NIV) in preterm with respiratory distress syndrome (RDS) to show that the illness and developmental condition were associated easily to obtained EIT measurements of ventilation homogeneity that may be employed during NIV [16]. Based on temporal and spatial data gathered from EIT assessments, contemporary EIT research in NIV on pediatric patients may be primarily divided into two groups:

#### 4.1.1 Noninvasive High‐Frequency Ventilation(NHFV)/Noninvasive High‐Frequency Oscillatory Ventilation (nHFOV)

High‐frequency ventilation allows for adequate ventilation normally over the functional residual capacity, and it is widely adopted by many neonatologists.

However, it is still unknown how the resulting oscillations are efficiently delivered to the alveoli. In a randomized crossover study comparing nasal continuous positive airway pressure (nCPAP) with noninvasive high‐frequency oscillatory ventilation (nHFOV) in thirty prone preterm infants, EIT was utilized to measure the oscillatory volumes during nHFOV, it observed ventilation transmit to the right lung and non‐gravity‐dependent lung [17].

#### 4.1.2 Nasal Continuous Positive Airway Pressure (nCPAP)

Nasal continuous positive airway pressure ventilation is commonly used for the treatment of respiratory failure in different children admitted to the NICU. Bhatia has described the regional patterns of EELV and VT during CPAP for setting the continuous distending pressure(CDP) in different increasing and decreasing pressure in 20 infants with mild respiratory distress syndrome who were less than 32 weeks gestation [18]. EIT has also been used to evaluate changes in EELV and VT during biphasic positive airway pressure (BiPAP) in some stable preterm infants In certain stable preterm newborns [19].

### 4.2 Mechanical Ventilation

Mechanical ventilation will inevitably lead to ventilator‐induced lung injury(VILI) affecting the prognosis of pediatric patients, more attention needs to be paid to the use of appropriate support pressure and tidal volume, and EIT helps to be cognizant of the optimization of child/ventilator interaction. EIT studies in MV on pediatric patients could be mainly categorized into the following studies:

#### 4.2.1 Synchronized Positive Pressure Inflations/Synchronized Volume‐Targeted Ventilation

In comparison to chaotic flow, synchronized pressure encourages laminar gas flow in the lung, which may be linked to a more uniform gas distribution and thus more protective [20]. VILI is a significant risk factor for bronchopulmonary dysplasia (BPD), the most common chronic respiratory disease in preterm infants, contributing to considerable psychosocial and economic burdens [21]. A prospective observational study that collected more than two thousand synchronous/inflations ventilation EIT images in 19 infants described synchronous mechanical ventilator lung inflation yielding more gravity dependence, with right to left ventilation and lung ventilation being more consistent than asynchronous inflation [22]. In preterm newborns, volume‐targeted ventilation (VTV) modes have been demonstrated to lower the risk of mortality, chronic lung disease, and short‐term morbidity by adjusting supplied pressure with the goal of maintaining a constant VT defined by the physician [23]. Meanwhile, EIT was possible in the stable to describe regional ventilation, mechanically ventilated, extremely preterm infant receiving synchronized intermittent positive ressure ventilation (SIPPV) + VTV [24]. EIT provides real‐time, individualized, and visualized guidance for both noninvasive and invasive mechanical ventilation for critically ill pediatric patients, and enabling continuous monitoring of pulmonary ventilation.

### 4.3 EIT Guided Positive End Expiratory Pressure Titration

The choice of suitable position end expiratory pressure (PEEP) is particularly important, however, there are no specific recommendations for setting PEEP for children. Inadequate PEEP may cause overdistension or atelectasis. In a series of 40 children under non‐thoracic surgery, investigate change increasing PEEP from 2 to 5 cmH2O during mechanical ventilation in children undergoing general anesthesia. [25]; The image monitoring conducted by EIT indicates evident signs of intertidal recruitment and derecruitment at PEEP levels of up to 5 cmH2O in pediatric patients. EIT also guides PEEP used in pediatric actue respiratory disease syndrome (PARDS), different individualized PEEP titration methods were proposed and often compared to each other [26]. EIT was effectively employed in a prospective interventional experiment including eight children who had early acute respiratory distress syndrome to show that titrating PEEP might prevent localized overdistention and collapse while improving global compliance and gas exchange [27]. Another study that looked at the differences between PEEP chosen with global dynamic respiratory system compliance and EIT in 12 children with moderate‐to‐severe pARDS came to the conclusion that PEEP chosen with the best respiratory system compliance (Crs) may not be worse than EIT‐guided regional ventilation in cases of moderate‐to‐severe pARDS because EIT provided information on ventilation distribution [28]. While there are similarities between PARDS and ARDS, data from adults cannot be applied directly to children since children's lungs have a greater elastin/collagen ratio and more chest wall compliance than adult lungs [29]. All in all, more research with a sizable sample of kids is necessary to validate the results.

### 4.4 EIT With Personalized Position

In spontaneously breathing neonates, EIT was used to observe how ventilation is distributed in the lung and how it is influenced by the breathing pattern or body position [30, 31, 32]. In ventilated neonates and pediatric patients, positioning is thought to be essential for maintaining adequate lung ventilation by maximizing oxygen delivery and gas exchange [33]. Previous studies advise that the prone position is beneficial. However, in earlier studies, researchers primarily used EIT monitoring in pediatric patients to observe ventilation distribution and measure regional impedance amplitudes and global inhomogeneity indices in healthy, spontaneously breathing infants and children under various personalized ventilation positions (such as prone or lateral positions) [12, 34, 35, 36, 37, 38]. But in a recent study in 2022, Long and his group reported successful application of EIT to evaluate pulmonary ventilation and perfusion matching in pARDS children [39]. EIT may be used as respiratory monitoring in ICU(intensive care unit), the pulmonary ventilation situation of children under different positions is also different, and EIT helps clinicians to observe the improvement of pulmonary ventilation of children at different positions.

### 4.5 EIT With Pneumothorax

The two main causes of newborn pneumothorax are respiratory distress syndrome (RDS) and premature delivery. A preterm neonate's lungs produce inadequate surfactant, which is the cause of RDS [40]. In an animal model, Costa et al. demonstrated that EIT could identify a pneumothorax with a sensitivity and specificity of 100% and 95%, respectively [41]. Undoubtedly a breakthrough for pediatric patients, pneumothorax, a potentially fatal consequence of intranasal continuous positive airway pressure or mechanical ventilation for preterm newborns [42]. While back in 2011, the alterations in lung impedance in a ventilated preterm newborn with unilateral pneumothorax were reported by Miedema [43]. Rahtu demonstrated changes in impedance and ventilation on the affected side in neonates with pneumothorax using EIT monitoring, even before the clinical diagnosis was made [44].

A study of neonatal RDS reported pneumothorax occurring in children with EIT monitoring strong spontaneous breathing efforts during neurally adjusted ventilatory assist (NAVA) real‐time monitoring helped clinical work early identification of such patients at venture of therapy complications, allowing more rapid detection of this undesirable event and earlier intervention [45]. EIT helps in assessing the progression or resolution of pneumothorax and may be able to help guide timely interventions to improve patient outcomes.

### 4.6 EIT With Pneumonia

A frequent cause of hospitalization for children is community‐acquired pneumonia (CAP). Children with CAP often recover clinically quickly after antibiotic therapy and usually do not require routine follow‐up chest X‐ray photography [46, 47, 48]. Mazzoni et al. and Karsten et al. investigated EIT as an additional instrument of choice for tracking lung ventilation distribution in pneumonia [49, 50]. In addition, during the global COVID‐19 pandemic, a report by Nascimento evaluated the ventilation distribution in six pediatric COVID‐19 patients using EIT and compared the findings with chest radiograph results. The study demonstrated that the EIT images were consistent with the patients' clinical manifestations, EIT revealed disparities in ventilation distribution between patients with pleural effusion and those exhibiting ground‐glass opacities, with the unevenness particularly pronounced in pediatric pleural effusion cases [51]. EIT can be used to assess the regional ventilation response to bronchodilators, providing insights into treatment efficacy in children with viral lower respiratory tract infections. In one study involving six children, EIT demonstrated improved ventilation distribution following bronchodilator administration [52].

### 4.7 EIT With Cystic Fibrosis and Asthma

The genetic ailment known as cystic fibrosis (CF) is an autosomal recessive condition that arises from mutations in the cystic fibrosis transmembrane conductance regulator gene, or cystic fibrosis transmembrane conductance regulator (CFTR). Numerous medical disorders affecting the pulmonary and other systems are common in people with CF. Spirometry is a vital tool for evaluating lung function in pediatric cystic fibrosis patients, yet many young children are not able to complete repetitive forced expiration maneuvers during lung function tests. Additionally, a considerable proportion of pediatric cystic fibrosis patients exhibit normal FEV1 values despite having underlying structural lung abnormalities. Compared with traditional diagnosis and monitoring image techniques, such as CT, EIT is a radio‐free tool for pediatrics to diagnose CF and response therapy, which is a new choice. The EIT technique correlated well with lung function data and radiological results [53, 54, 55, 56, 57]. A 2018 observational study used EIT to monitor CF pediatric patients, which provides premier proof of EIT in CF patients by correlating EIT‐derived measures of pulmonary function with spirometry, the gold standard for lung function assessment [58]. Similarly, a study demonstrates that EIT effectively complements spirometry and body plethysmography in children with PCD by addressing key limitations: EIT detected ventilation inhomogeneity in all patients, whereas spirometry (FEV₁) only identified abnormalities in 41.7% (5/12). Crucially, EIT provided regional localization (e.g., highest impairment in the right dorsal quadrant), which global measures like plethysmography's RV/TLC cannot offer. Furthermore, EIT requires no patient collaboration, making it particularly feasible for young children compared to effort‐dependent techniques like spirometry. EIT serves as a valuable complementary tool for characterizing lung impairment in PCD [59]. Besides, EIT can recognize air trapping, a characteristic of CF, and offer details on the air trapping [60]. To show the viability of EIT for visual diagnosis of asthma, EIT measurements were carried out during pulmonary function test in 58 children with asthma utilizing average flow‐volume (FV) loops that created for patients with pathologic spirometry. It has a good relationship with spirometry. A study comparing EIT with traditional lung function tests demonstrated that EIT can identify lung function heterogeneity and assess its extent [61]. In FV loops obtained from EIT, positive bronchospasmolysis can be detected [62]. In conclusion, EIT shows a high potential for pulmonary function testing for pediatric patients. It offers a new option for patients who need to test lung function but do not fit well.

### 4.8 EIT With ECMO

Extracorporeal membrane oxygenation (ECMO) is a broad term used to maintain a neonate, child, or adult's heart and/or lungs. When it comes to ECMO, pediatric patients and adult patients differ greatly. During the usage of ECMO in severe children, knowledge of the distribution of pulmonary ventilation is beneficial for help setting ventilator parameters. A research assessed the ventilation distribution at ultraprotective ventilator settings for pediatric patients receiving venovenous ECMO [63]. In this article, EIT facilitated critical support, including PEEP titration and dynamic lung ventilation monitoring, during ECMO treatment in this child. Further insights are necessary regarding pulmonary status during ECMO, encompassing guidance for effective pulmonary rehabilitation. EIT has immeasurable potential in guiding clinical work in many different areas of ventilator setting for pediatric patients on ECMO.

### 4.9 EIT With Anesthesia

Unlike adults, children do not cooperate very well when they get medical examinations. Especially for some longer time examinations, the doctor will give some sedative drugs appropriately. In a study of 2022, twenty‐five children were included, they got MRI under deep sedation and kept spontaneously breathing. As the EIT shows, deep sedation resulted in a significant decrease in lung ventilation without affecting overall ventilation homogeneity [64]. A recent case reported a preterm infant who experienced a drawn‐out chest wall stiffness after using fentanyl, the clinicians used EIT proved minimal tidal volumes and thereby, which is useful for rectify placement of the endotracheal tube accurately [65]. In a prospective observational survey of 20 children under 5 years old, ventilation redistribution from the dorsal side to the ventral side after initiation of mechanical ventilation was monitored using EIT and conclude the percentage of ventilation distribution in the posterior region decreased 5%. With the recovery of spontaneous breathing function, the dorsal ventilatory function was recovered [66]. Wilsterman demonstrated that in some children with pARDS, neuromuscular blockade had no effect on the distribution of VT and regional lung filling characteristics [67]. Some studies used EIT to confirm the gradual reduction of ventilation in non‐thoracic surgery during controlled mechanical ventilation, especially in the dorsal region, and the visual characteristics of EIT confirmed the previous hypothesis that progressive atelectasis is mainly focused on the gravity‐dependent part [5, 68]. These demonstrate how effective EIT may be as a monitoring technique for breathing optimization during clinical anesthesia.

### 4.10 EIT Monitor Thoracic Sugery

Postoperative pulmonary complications (PPCs) are a relatively significant risk following thoracic surgery due to the impairment of postoperative respiratory function. In addition to having a huge clinical and financial impact on the number of fatalities that follow thoracic surgery, PPCs can cause lengthy hospital stays and need admissions to critical care units. Therefore, it is very necessary to monitor the lung condition of children after chest surgery in real time. In 2014, EIT was used to analyze changes in regional lung ventilation in pediatric patients after cardiac surgery [4]. After heart surgery, EIT was utilized to examine changes in regional lung ventilation in pediatric patients. Currently, there are still only a few studies on the thoracic using EIT after chidren's chest surgery. It is limited by the size of the belt and the pack after chest surgery hinders the bonding of the belt. By providing a dynamic and detailed view of lung function, EIT can enhance recovery and improve patient outcomes after surgery.

### 4.11 EIT With Perfusion

The Impedance property of blood and air is different. EIT has also been explored to evaluate the spatial distribution of perfusion. Now, there are two different methods for EIT to measure pulmonary perfusion, one approach is to inject hypertonic saline solution for a short time(< 2 s) through the central vein, and the other is to directly describe the volatility of blood flow through the lungs by algorithms [69, 70, 71, 72, 73]. An EIT perfusion graphic with abnormal pulsatility due to pulmonary thrombosis was observed in a COVID‐19 adolescent in a case report [74]. Meanwhile, another case study assessed pulmonary blood flow in a child with pARDS using hypertonic saline [40] The purpose of the two different methods is to achieve real‐time bedside monitoring of pulmonary blood flow, and the real noninvasive way is the consistent goal of the development direction of EIT monitoring of pulmonary perfusion. EIT's noninvasive nature and ability to provide continuous monitoring make it a valuable tool for critically ill patients who require ongoing evaluation of lung perfusion.

### 4.12 Miscellaneous

#### 4.12.1 Sickle Cell Disease

Sickle cell disease (SCD) is a hematological disease caused by abnormal hemoglobin. It always leads to pulmonary illness that are closely associated with mortality. Research proved that restrictive ventilatory problems affected most people with SCD [75, 76]. In 2023, Rein used EIT to demonstrate that the pattern of lung dysfunction in the young SCD patients is restricting, with FEV1/FVC showing normal values while FEV1 and total lung capacity(TLC) were frequently pathologically decreased [77].

#### 4.12.2 Pulmonary Langerhans Cell Histiocytosis(PLCH)

A case study of 2018 reported a 2‐year‐old boy, with terrible PLCH, the chest radiography revealed cystic variations, the lung condition was poor, the oxygenation index was very poor, experienced many invasive operations and ventilation mode adjustments and a long time of treatment, eventually children with a good prognosis, EIT provides valuable guidance about regional ventilation for various clinical treatment. This intervention immediately improved right‐posterior ventilation to 39% of tidal volume and reduced global hyperinflation (impedance drop from 5626 to 1150) [78]. EIT offers unique insights into regional ventilation distribution, enabling real‐time and gave positive feedback allowing continuation of strategies.

#### 4.12.3 Tracheobronchial Foreign Body(FBA)

Foreign body aspiration is a frequent problem in pediatrics that can lead to different problems that has to be treated right soon to prevent complications and irreparable lung injury [79]. EIT helps a team Identify a bronchial foreign body in a nineteen‐month‐old boy, it's a convenient detection tool compared to visual bronchoscopy when faced with clinical diagnose‐clue suspicion [80]. Table 2 list some key studies about EIT for validating the previously discussed clinical applications is delineated.

**Table 2:** Key studies on clinical application of Neonatal and Pediatrics patients in EIT.

Study publication	Study type	Intervention	Population	EIT parameter	References
Thomas/2009	Cross‐sectional group comparison study	EIT as an observational tool	17 healthy term‐born /15 preterm infants	Global inhomogeneity	[3]
Bronagh/2020	Observational study	Chest physiotherapy	17 participants received CPT/43 not	EELV Geometric center Global inhomogeneity	[15]
Isabel/2019	Prospective observational study	EIT guide‐PEEP	8 children with early acute respiratory distress syndrome (< 72 h)	Overdistension and collapse (OD/CL)	[27]
Lupton /2017	Prospective observational study	Prone Positioning	12 children	Global inhomogeneity	[37]
M Miedema/2011	Case report	EIT as an observational tool	1 Preterm Infant	Asymmetrical distribution	[43]
Sylvia/2016	Prospective controlled trial	Cystic fibrosis	11 patients with CF/11 lung healthy controls	Lung function test	[53]
Thomas /2022	Prospective observational trial	Procedural deep sedation	25 children	Global inhomogeneity	[64]
Flávia /2022	case report	Pulmonary thrombosis	15‐year‐old girl	pulmonary perfusion	[74]
Robert /2021	case report	Tracheobronchial foreign body aspiration	19‐month‐old boy	Global ventilation	[80]

## 5 Limitation and Future Directions and Conclusion

As a bedside, noninvasive, non‐radiative monitoring tool, the EIT improves the chest model of newborn and pediatric patients and describes the pulmonary ventilation blood flow in neonatal and pediatric patients more accurately. EIT also has several limitations in clinical use. First, EIT systems face challenges in achieving high spatial resolution and signal stability, particularly in small patients such as neonates and children. Second, maintaining consistent real‐time monitoring is challenging due to factors such as patient movement, electrode displacement, and variations in electrode contact quality. Lastly, in our study, the evidence base for EIT in pediatrics is limited, with most studies being case reports, and some involve relatively small sample sizes, which may limit their reliability. However, these studies provide valuable insights for clinicians applying EIT at the bedside to monitor pulmonary in children. The potential for applicability in newborn and pediatric patients is enormous, regardless of the setting—general clinical ward or critical care unit—due to the contrasts between these patient populations and adult patients. In addition to the routine monitoring performed throughout the use of ventilators in critically ill patients, EIT serves as a valuable tool for detecting conditions such as pneumothorax and assessing lung function. Its utility as a chest monitoring modality ultimately depends on the clinician's intended application to support specific clinical tasks. EIT may become a ubiquitous imaging modality, but large‐scale, multicenter investigations are required to assess its clinical efficacy in pediatric patients who are critically sick.

## Author Contributions

**Xuelian Yang:** conceptualization, methodology, validation, investigation, writing – review and editing, writing – original draft, formal analysis, data curation. **Gelan Miao:** methodology, data curation, formal analysis, writing – original draft. **Chaobing Yang:** methodology, data curation, formal analysis. **Li Liu:** Writing – review and editing. **Xianying Lei:** Writing – review and editing, Funding acquisition, Resources.

## Conflicts of Interest

The authors declare no conflicts of interest.

## Transparency Statement

The lead author Xianying Lei affirms that this manuscript is an honest, accurate, and transparent account of the study being reported; that no important aspects of the study have been omitted; and that any discrepancies from the study as planned (and, if relevant, registered) have been explained.

## Data Availability

Data sharing not applicable to this article as no datasets were generated or analysed during the current study. All authors have read and approved the final version of the manuscript Xianying Lei had full access to all of the data in this study and takes complete responsibility for the integrity of the data and the accuracy of the data analysis.
